# Role of post-chemotherapy radiation in the management of children and adolescents with primary advanced malignant mediastinal germ cell tumors

**DOI:** 10.1371/journal.pone.0183219

**Published:** 2017-08-16

**Authors:** Junting Huang, Yuting Tan, Zijun Zhen, Suying Lu, Feifei Sun, Jia Zhu, Juan Wang, Ru Liao, Xiaofei Sun

**Affiliations:** 1 Department of Pediatric Oncology, State Key Laboratory of Oncology in South China, Collaborative Innovation Center for Cancer Medicine, Sun Yat-Sen University Cancer Center, Guangzhou, Guangdong, P. R. China; 2 Department of Radiation Oncology, State Key Laboratory of Oncology in South China, Collaborative Innovation Center for Cancer Medicine, Sun Yat-Sen University Cancer Center, Guangzhou, Guangdong, P. R. China; Universidad de Navarra, SPAIN

## Abstract

**Objective:**

Primary malignant mediastinal germ cell tumors (MMGCTs) are rare in children and adolescents and have a poorer prognosis than their gonadal counterparts. We report a single institutional experience of a 10-year period of primary advanced MMGCTs treated with chemotherapy, followed by radiotherapy in those who had residual mass.

**Methods:**

Children and adolescents with primary advanced MMGCTs between 2005 and 2014 were identified from the Cancer Center, Sun Yat-Sen University. Medical records were reviewed for clinicopathological characteristics, treatments, and outcomes.

**Results:**

Twenty-four children and adolescents with either stage III or IV primary advanced MMGCTs met the inclusion criteria. There were 23 males and one female with a median age of 16 (range 10–18). Seven cases were seminomas (29.2%); four (16.7%) yolk sac tumors (YST); three (12.5%) choriocarcinomas; and ten (41.6%) nonteratomatous combined germ cell tumors (CGCTs). All patients were treated with first-line cisplatin-based chemotherapy regimens (PEB: 19, VIP: 5). Thirteen (54.2%) and Twelve (50%) patients received surgery and radiotherapy, respectively. With a median follow-up of 46.2 months (range 9.6–124.8 months), a total of five (20%) patients died of disease progression; the five-year overall survival (OS) and disease-free survival (DFS) rates were 82.3% and 64.9%, respectively.—Seven patients with seminoma GCTs received post-chemotherapy irradiation were alive with sustained CR (5-year OS and DFS, 100%, respectively). Five patients with NSGCTs were administered irradiation and one relapsed 35 months later and died of metastasis (5-year OS, 100%; 5-year DFS 66.7%). Univariate analysis identified histology and stage were prognostic factors.

**Conclusion:**

Multimodality treatment approach of chemotherapy followed by radiation consolidation ensured long-term survival in primary advanced MMGCTs. Further research is warranted to improve the prognosis of children with primary advanced MMGCTs.

## Introduction

The mediastinal germ cell tumors (GCTs) are rare in children and adolescents, comprising 3–6% of all GCTs diagnosed among children and adolescents younger than 18 years of age[1,2,3]. As the most common extragonadal GCTs (EGCTs), these tumors are considered to arise from the abnormal migration of germ cells during embryogenesis[4]. Since the adoption of cisplatin-based multiagent chemotherapy in the 1970s, the survival rate of GCT patients has improved markedly to 80% or higher[5,6]. Despite primary malignant mediastinal germ cell tumors (MMGCTs) share similar histology, serologic tumor markers, as well as genetic abnormalities with their gonadal counterparts, they have an inferior prognosis for survival than those with primary tumors of the gonads: the five-year survival rate ranges from 40% to 60%[7,8,9,10].

Even though treatment of gonadal GCTs is well established, primary MMGCTs remain a challenge for oncologists. Chemotherapy alone seldom contributes to satisfactory outcomes with five-year survival range from 30%-60%[4,7,8,11]. Surgical resection of residual mass after chemotherapy plays an important role in the management of patients with primary MMGCTs[11]. However, primary MMGCTs infiltrating vital organs and vessels are contradictions to salvage surgery, radiotherapy can be used in this setting and has showed significantly improved survival for adult patients[12].

Owing to the rarity of primary MMGCTs in children and adolescents, limited data about radiation therapy is available for pediatric oncologists. In this context, we performed a retrospective analysis of the recent experience with primary advanced MMGCTs at Cancer Center of the Sun Yat-Sen University.

## Materials and methods

### Ethics statement

This study was approved by the Medical Ethics Committee of Sun Yat-Sen University Cancer Center. The need for informed consent was waived by the Medical Ethics Committee because the study was an observational, retrospective study using a database from which the patients’ identification information had been removed.

### Patients

We retrieved the clinical database to identify children and adolescents aged 18 years or younger with primary advanced MMGCTs who had been referred to the Cancer Center, Sun Yat-Sen University from 2005 through 2014. A primary malignant mediastinal germ cell tumor is defined as occurring in a mediastinum location without evidence of gonadal mass detectable by diagnostic imaging examination. Histologically, the presence of malignant elements within the tumor was required, including seminoma, yolk sac tuomors (YST), embryonic carcinoma (EC), choriocarcinoma, nonteratomatous combined germ cell tumors (CGCTs) or teratomas with additional malignant components[13,14].

Detailed information such as gender, age, symptoms, medical history, location of primary tumor; tumor markers including serum alpha fetoprotein (AFP) and β-subunit human chorionic gonadotrophin (β-HCG), surgery or radiotherapy, chemotherapy regimen, response, and follow-up outcome were collected.

### Staging

Staging was based on the Children’s Oncology Group (COG) Staging System for GCTs as follows: stage I—complete resection at any site with negative margins; stage II—microscopic residual, with lymph nodes negative; stage III—lymph node involvement, gross residual disease, or biopsy only; stage IV—distant metastases, including liver, brain, bone, or lung[14,15].

### Surgery

Surgical resection of the primary tumor was administered when feasible, followed by four to six cycles of chemotherapy consisting of cisplatin. If the tumor was not optimally resectable at diagnosis, biopsy specimen was obtained for histological examination. Re-evaluation for resection was considered after two to four courses of preoperative cisplatin-based multiagent chemotherapy. Complete resection was defined as the absence of macroscopic or microscopic residual tumor and postoperative normal serologic tumor markers, and incomplete resection was defined as the presence of macroscopic or microscopic residual tumor. For patients who received post-chemotherapy surgery with residual disease, two additional courses of chemotherapy were given.

### Chemotherapy

The commonly used chemotherapeutic schedules were PEB (cisplatin 20 mg/m^2^/d on Days 1–5, etoposide 100 mg/m^2^/d on Days 1–5, bleomycin 15 mg/m^2^/d on Days 1), VIP (etoposide 100 mg/m^2^/d on Days 1–4, ifosfamide 1.5 g/m^2^/d on Days 1–4, cisplatin 25 mg/m^2^/d on Days 1–4). Chemotherapy was given with 21-day intervals.

### Radiotherapy

Radiotherapy was carried out with a linear accelerator using 6–8 MV X-rays at 1.8–2.0 Gy per fraction per day, 5 days per week, including techniques of conventional two-dimensional radiotherapy (2DRT) for three patients, three-dimensional conformal radiotherapy (3DCRT) for seven patients, and intensity modulated radiation therapy (IMRT) for two patients. Median dose of 36 Gy (range 30 Gy-50 Gy) and 50 Gy (range 45 Gy-60 Gy) were given to patients with seminoma germ cell tumors (SGCTs) and nonseminomous germ cell tumors (NSGCTs), respectively. The treatment volume was determined by pretreatment radiographic studies.

### Evaluation of efficacy and toxicity

For chemotherapy, response was evaluated every two courses according to the Response Evaluation Criteria in Solid Tumors (revised RECIST) 1.1[16]. Complete response was defined as no detection of tumor and normalized tumor marker. All toxicity results related to chemotherapy or radiotherapy were graded using the National Cancer Institute Common Terminology Criteria for Adverse Events version 4[17].

### Statistical analysis

Disease-free survival (DFS) was calculated from the date of pathological diagnosis of primary MMGCT to disease progression, relapse, and death from any cause or last follow-up. Overall survival was calculated from the date of diagnosis until death or the date of last visit. Statistical analysis was performed using SPSS 23.0 software. Survival curves were estimated with the Kaplan-Meier method. Univariate comparisons were made using the log rank test. Multivariate analysis was made using Cox proportional hazards regression model. Two-tailed P-value < 0.05 was considered to be statistically significant.

## Results

### Patient characteristics

Thirty-one (7.26%) of 427 children and adolescents with GCTs were diagnosed with primary MGCTs at Sun Yat-Sen University Cancer Center between 2005 and 2014. Nine patients who were diagnosed with mature teratoma were excluded. Median age at diagnosis was 16 (range 10–18). The male-to-female ratio was 23:1. All of the primary tumors located at the anterior mediastinum. At the time of diagnosis, 23 (95.8%) patients complained of nonspecific symptoms, such as chest pain (16, 66.7%), cough (12, 50%), dyspnea (9, 37.5%), and fever (7, 29.2%). Two patients (8.3%) had superior vena caval obstruction (SVCO). AFP and β-HCG were measured preoperatively in 22 patients. Of the 24 patients, 7 (29.2%) had SGCTs and 17 (70.8%) had NSGCTs according to the results of histological and serum tumor marker evaluations. Stage III or IV disease accounted for 20 (83.3%) and four (16.7%) patients, respectively. Clinical characteristics are presented in Table 1.

**Table 1:** Patient characteristics(N = 24).

Characteristic	No. of patients	%
Age (yrs)		
≤15	10	41.7
>15	14	59.3
Gender		
Male	23	95.8
Female	1	4.2
Symptoms		
Symptomatic	23	95.8
chest pain	16	66.7
cough	12	50.0
dyspnea	9	37.5
fever	7	29.2
SVCO	2	8.3
Asymptomatic	1	4.2
Location		
Anterior mediastinum	24	100
Size (cm)		
<10	9	37.5
≥10	15	62.5
Histology		
Seminoma	7	29.2
Nonseminoma	17	70.8
CGCTs	10	41.7
YST	4	16.7
choriocarcinoma	3	12.5
Tumor marker status		
AFP		
Tested	22	91.7
Elevated at diagnosis	14	58.3
β-HCG		
Tested	22	91.7
Elevated at diagnosis	16	66.7
Stage		
Stage III	20	83.3
Stage IV	4	16.7

AFP, alpha fetoprotein; β-HCG, β-subunit human chorionic gonadotrophin; SVCO, superior vena caval obstruction.

### Treatments and response

First-line cisplatin-based multidrug chemotherapy (19 PEB, 5 VIP) was given to all patients as mainstay modality throughout treatment. With the first-line treatment strategy, five patients were treated with chemotherapy only. 15 patients were given neoadjuvant chemotherapy, and four patients received adjuvant chemotherapy. A median of four courses of chemotherapy were administered. Overall, three (12.5%) of 24 patients had a complete response (CR), 17 (70.8%) partial response (PR), three (12.5%) stable disease (SD) and one (4.2%) progression disease (PD), respectively. Of the five patients treated with VIP, one developed Grade III leucopenia and two developed Grade IV leucopenia combined with febrile neutropenia (FN) or peritonitis. In the PEB group, eight patients had Grade III (n = 6) and IV (n = 2) myelosuppression and three patients had Grade III gastrointestinal toxicity.

A total of four patients were eligible for primary resection of the tumor according to preoperative imaging. Four surgical candidates who were performed initial tumor resection did not achieved complete resection or normalized tumor markers after surgery. For nine patients with unresectable disease at diagnosis, two to four courses of chemotherapy were given to shrink the tumor. Four (44.4%) acquired complete resection and normalized tumor markers, while five remained a residual mediastinal mass.

12 patients were administered radiotherapy without concurrent chemotherapy: six following chemotherapy alone; six following the combination of chemotherapy and surgery. Six patients whose tumor resection was not feasible at diagnosis received four to six cycles of chemotherapy (5 PEB, 1 VIP). One patient with pre-chemotherapy bulky mass achieved complete response (CR) continue to receive adjuvant radiotherapy. Another five patients who did not meet the criteria of re-evaluation resectability after PR to neoadjuvant chemotherapy received definitive irradiation. Two patients with pretreatment bulky tumor were given -adjuvant irradiation after preoperative chemotherapy and complete resection. Four patients with postoperative residuals were given irradiation. Grade I and grade II acute esophagitis were reported in one patients respectively. No patient developed ≥ grade III toxicity. The details of first-line treatment modality are shown in Table 2.

**Table 2:** Details of patients(N = 24).

Case	Age (yrs) /gender	Histology	AFP at onset (ng/ml)	β-HCG at onset (mIU/ml)	First-line treatment modality	Stage	Chemotherapy regimen	Postoperative AFP (ng/ml)	Postoperative β-HCG (mIU/ml)	Irradiation/ dose (Gy)	Outcome
1	14/M	Choriocarcinoma	Normal	48574	C	IV	PEB			-	DOD
2	14/M	Seminoma	Normal	28	C→R	III	PEB			2DRT/36	Alive
3	18/M	Seminoma	Normal	30	C→R	III	PEB			2DRT/50	Alive
4	18/M	Choriocarcinoma	Normal	628	C→S	III	PEB	Normal	103	-	DOD
5	16/M	CGCT	17703	540	C	IV	VIP			-	DOD
6	15/M	CGCT	47798	5612	C	III	PEB			-	DOD
7	18/M	CGCT	6252	Normal	C→S→R	III	PEB	Normal	Normal	3DCRT/52	DOD
8	15/M	CGCT	27585	47	C	IV	PEB			-	Alive
9	18/M	CGCT	8552	5978	C→S→R	III	PEB	Normal	Normal	3DCRT/50	Alive
10	14/M	Seminoma	-	-	S→C→R	III	PEB	-	-	3DCRT/40	Alive
11	18/M	Seminoma	Normal	64	C→R	III	PEB			2DRT/36	Alive
12	15/M	Seminoma	Normal	Normal	C→R	III	PEB			3DCRT/36	Alive
13	15/M	Seminoma	Normal	27	C→R	III	PEB			3DCRT/38.6	Alive
14	18/M	Seminoma	Normal	1103	C→S→R	III	PEB	Normal	Normal	3DCRT/36	Alive
15	17/M	YST	9546	Normal	C→S	III	VIP	Normal	Normal	-	LOF
16	16/M	CGCT	303	48	C	III	PEB			-	Alive
17	14/M	CGCT	1654	Normal	S→C→R	III	PEB	Normal	Normal	3DCRT/45	Alive
18	10/F	CGCT	104481	106	C→S	IV	PEB	Normal	Normal	-	Alive
19	16/M	CGCT	45857	Normal	S→C→R	III	PEB	3972	Normal	IMRT/50	Alive
20	14/M	Choriocarcinoma	4921	931225	C→S	III	PEB	Normal	Normal	-	Alive
21	17/M	YST	17732	Normal	C→R	III	VIP			IMRT/60	Alive
22	14/M	YST	65606	Normal	C→S	III	VIP	Normal	Normal	-	Alive
23	18/M	YST	52406	Normal	C→S	III	VIP	3339	Normal	-	LOF
24	14/M	CGCT	-	-	S→C	III	PEB	72	Normal	-	Alive

M, male; F, female; SVCO, superior vena caval obstruction; CGCT, combined germ cell tumors; YST, yolk sac tumor; AFP, alpha fetoprotein; β-HCG, β-subunit human chorionic gonadotrophin; –, Not performed, C, chemotherapy; S, surgery; R, radiotherapy; PEB, bleomycin, etoposide, and cisplatin; VIP, etoposide, ifosfamide, cisplatin; 2DRT, two-dimensional radiotherapy; 3DCRT, three-dimensional conformal radiotherapy; IMRT, intensity modulated radiation therapy; DOD, dead of disease; LOF, loss of follow-up.

## Outcome

**Fig 1:**
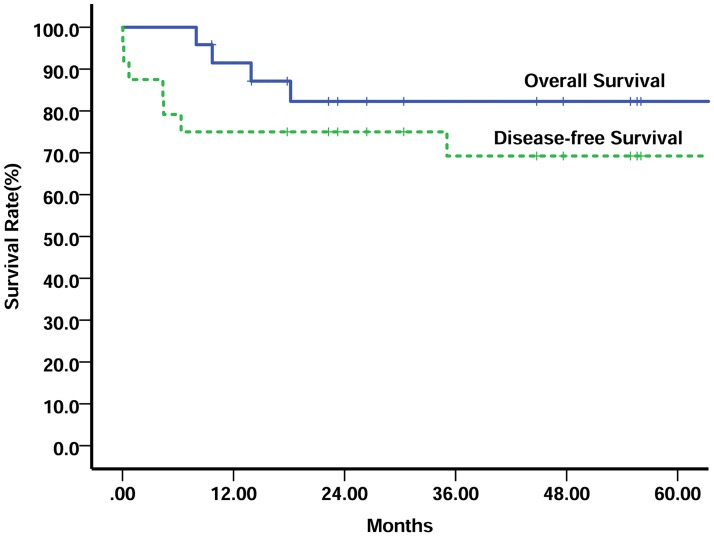
Overall survival and disease-free survival of the entire group.

**Fig 2:**
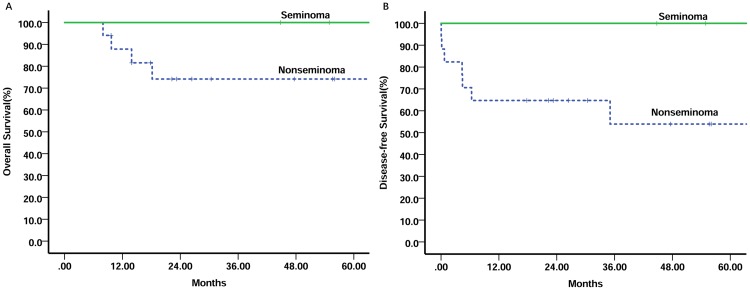
Kaplan-Meier overall survival (A p = 0.032) and disease-free survival (B p = 0.045) stratified by histology.

With a median follow-up of 46.2 months (range 9.6–124.8 months), a total of 7 patients relapsed: three patients died of distant metastases; two patients died of local progression; two patients censored after local recurrence. The remaining 16 patients were alive with no evidence of disease continuously. The overall survival (OS) and disease–free survival (DFS) of the entire group at 5 years were 82.3% and 64.9%, respectively (Fig 1). The 2-year OS and DFS of patients with seminoma were 100.0% and 100.0% versus 74.2% and 64.7% for patients with nonseminoma, respectively (P = 0.032, P = 0.043) (Fig 2). Three of five local failure was observed in patients who received perioperative chemotherapy and surgical resection. Including surgery in the primary treatment regimen did not appear to have an impact on survival outcome for any of the patients. None of the patients who received adjuvant radiotherapy had local recurrence. Seven patients with seminoma GCTs received post-chemotherapy irradiation were alive with sustained CR (5-year OS and DFS, 100%, respectively). Five patients with NSGCTs were administered irradiation and one relapsed 35 months later and died of metastasis (5-year OS, 100%; 5-year DFS 66.7%). The univariate analysis of prognostic factors on survival is shown in Table 3. Stage IV and nonseminoma were predictive of adverse prognosis.

**Table 3:** Univariate analysis of survival.

Variables	No. of patients	2-years OS (%)	P value	2-years DFS (%)	P value
Age (yrs)					
≤15	12	82.5	0.575	83.3	0.237
>15	12	82.5		66.7	
Size (cm)					
<10	9	77.8	0.999	66.7	0.810
≥10	15	85.6		80.0	
Histology					
Seminoma	7	100	0.032	100	0.045
Nonseminoma	17	74.2		64.7	
AFP (ng/mL)					
≤10,000	14	84.6	0.631	78.6	0.517
>10,000	8	72.9		62.5	
Not tested	2	100		100	
β-HCG (mIU/ml)					
≤25	8	100	0.543	75.0	0.658
>25	14	71.4		71.4	
Not tested	2	100		100	
Stage					
Stage III	20	88.8	0.025	80.0	0.177
Stage IV	4	50.0		50.0	
First-line chemotherapy					
PEB	19	83.9	0.572	77.7	0.029
VIP	5	80.0		40.0	
First-line Surgery					
Yes	13	91.7	0.655	76.9	0.974
No	11	72.7		72.7	
First-line treatment modality					
≤2 treatments	17	80.5	0.709	70.6	0.779
3 treatments	7	85.7		85.7	

AFP, alpha fetoprotein; β-HCG, β-subunit human chorionic gonadotrophin; PEB, bleomycin, etoposide, and cisplatin; VIP, etoposide, ifosfamide, cisplatin; OS, overall survival; DFS, disease-free survival;

## Discussion

Germ cell tumors (GCTs) predominantly arise from the gonads (both ovary and testis) and occasionally midline structures[18]. The mediastinum is the most common extragonadal primary tumor site[4]. Mediastinal germ cell tumors (MGCTs) account for 19%–25% of all mediastinal tumors and 3%–6% of all GCTs in pediatric age[1,2,3,19,20]. In our experience, the mediastinal site represents 7.26% of all GCTs registered from 2005 to 2014 at Sun Yat-Sen University Cancer Center. This percentage is a little higher than data reported in the literature because our hospital is a referral center and received complicated patients-[1,2,3].

As sharing similar histology with their gonadal counterparts, it is reasonable to extrapolate from the treatment of male testicular GCTs. Platinum-containing chemotherapy is the mainstay treatment of advanced GCTs. In our study, the predominant gender is male and all cases had stage III or IV diseases. In the current study, all patients receiving first-line cisplatin-based chemotherapy obtained 95.8% ORR. However, due to the advanced stage of our cases, CR was just achieved in 12.5% patients, which is lower than other reported results[21,22]. Response rate to salvage second-line chemotherapy was disappointing. Sustained CR rates were ranging from 24%-25% with standard conventional-dose regimens VIP, 38%-47% with TIP, or 31%-35% with high-dose chemotherapy and autologous stem-cell rescue[23,24,25,26].

It is estimated that 30%–88% of the residual mass contained viable cells[8,11,27]. Patients who did not achieve sustained CR got relapsed sooner or later. Surgical resection plays an important role in nonseminomous GCTs, which serves to remove chemotherapy-resistant disease, assess the pathological response to chemotherapy and guide subsequent treatment. Vuky et al. reported that complete resection of all gross residual disease was achieved in 84% patients with nonseminoma arising from the mediastinum after first-line or second-line chemotherapy[28]. In our study, three surgical candidates with NSGCTs who were performed initial tumor resection did not achieved complete resection or postoperative normalized tumor markes while the other three (37.5%) patients given surgery following first-line chemotherapy acquired complete resection and normalized tumor markers. Schneider et al. reported that the prognosis is favorable with a therapeutic strategy of delayed resection after preoperative chemotherapy in most children. Patients who underwent complete resection, had superior EFS (0.94±0.06 vs 0.42±0.33; P < .002) compared to those with incomplete resection[3].

In seminoma GCTs cases of residual tumor >3 cm and tumor marker levels that are normal, a PET scan is recommended to assess whether there are residual viable tumor cells[29]. However, a PET scan has a high false-positive incidence with regard to mediastinal malignancies[30]. SGCTs are sensitive to radiation. Yet there are relatively few published reports of radiation therapy for children and adolescents with primary MMGCTs[1]. On reviewing the literature, radiotherapy administered to paitents was mainly conventional two-dimensional technique reported in the 1980s and 1990s[31,32]. Novel radiation techniques, 3DCRT and IMRT have replaced 2DCRT since 2000s with higher dose deliver and lower treatment-related toxicity. In our series, seven patients with SGCTs received post-chemotherapy irradiation were alive with sustained CR (5-year OS and DFS, 100%, respectively). No patient developed ≥ grade III toxicity. Seldom researches suggest radiotherapy in the management of patients with NSGCTs. Recently, Wang et al. reported radiotherapy in a chemotherapy-based treatment regimen could significantly reduce local recurrence (77.3% vs. 38.5%, P = 0.003) and improve survival (68.2% vs. 38.5%, P = 0.043) of malignant mediastinal NSGCT patients[12]. In our series, five patients with NSGCTs were administered irradiation and one relapsed 35 months later and died of metastasis (5-year OS, 100%; 5-year DFS 66.7%).

The optimum dose and field size for radiotherapy in MMGCTs has not been well established. A dose of 3000 cGy should be adequate to control SGCTs within the mediastinum, and a boost treatment of 600 cGy may be used for bulky disease[31,33]. In our study, all seven patients with seminoma were administered with doses greater than 36.00 Gy and no one relapsed. NSGCTs are less radiosensitive compared with seminoma[34,35]. Yalcın reported three of four children who received a radiation dose below 40 Gy died of disease progression[1]. Wang reported five patients had significantly poor outcomes with doses below 45 Gy[12]. Renata et al. reported administering 45 Gy to one patient with residual tumor that was later resected, with no viable malignant cells found in the specimen[36]. In our series, the five patients with NSGCTs received ≥ 45 Gy irradiation and no one experienced local recurrence.

In 2DCRT era, some investigators recommended the inclusion of lower cervical and supraclavicular while others depended on whether the tumor was beyond the mediastinum[12,31,37,38]. In the present study, none of the three patients who were administered 2DRT had lower cervical and supraclavicular irradiated as disease did not spread to these regions. In post-2DCRT era, the irradiation field is defined as planning target volume (PTV), including clinical target volume (CTV) with a margin of 5 mm for set-up error. CTV is defined as the gross/residual tumor volume visible on pretreatment images with 5–20 mm margin according to the practice of each center.

Most patients with primary MMGCTs were symptomatic at the time of initial diagnosis due to the compression and/or invasion into the adjacent structures by the mass[19,39]. Chest pain, coughing and respiratory distress were the most common clinical symptoms[3,37]. In our cases, primary MMGCTs invariably occupied the anterior compartment. All but one patient presented with unspecific symptoms such as chest pain (66.7%), cough (50%) and dyspnea (37.5%). SVCO syndrome was observed in two patients. Radiologic examination indicated tumor resection was not feasible in 83.3% patients with lesions invading mediastinal organs or adhesion to great vessels at diagnosis, which could lead to an inadequate resection or potentially life-threatening complications during surgery.

The risk classification system for pediatric extracranial GCTs presented by Frazier et al. identified a group of patients age > 11 years with either stage III to IV extragonadal tumors or stage IV ovarian tumors with predicted LTDF survival < 70%. In our series, all but one patients aged 10 fell in this group[40]. The five-year OS and DFS rates were 82.3% and 64.9%, respectively. In the TGM-95 study, The five-year OS and EFS were 82.5% and 68%, respectively[41]. It should be noted that 93.8% patients received neoadjuvant chemotherapy before surgery and 80% patients got microscopic complete resection in the French study compared with 44.4% complete resection of our series. A total of five patients died of disease progression, all of which were diagnosed with NSGCTs. And three of the five patients only received chemotherapy, which suggests platinum-based chemotherapy alone is not effectively enough for advanced stged NSGCTs. Univariate analysis identified that stage and histology were prognostic factors. Receiving surgery in the first-line treatment modality was not significantly associated with increased survival because some patients received resection in the second or more line therapy. What’s more, two local recurrence and one local progression occurred in patients who underwent surgery while none of the patients who received adjuvant radiotherapy had local failure. It is not the timing of surgery but complete resection matters. In case of residual mass after surgical resection, salvage radiotherapy may be an effective option.

The treatment of GCTs has evolved substantially in the last three decades. Although it is a single-center retrospective study with limited number of patients, we selected a group of children and adolescents of the last decade treated with relatively standard first-line regiment consisting of cisplatin-based multidrug chemotherapy and/or surgery and/or radiation. Our findings suggested that radiotherapy following chemotherapy in the first-line treatment strategy is effective in SGCTs arising from the mediastinum with more safety and less toxic, and may spare extensive surgery with high risk of incomplete resection and complications. As for advanced mediastinal NSGCTs, although the OS for those who received radiotherapy reached 100%, this is based on five patients. Surgical resection is irreplaceable and delayed surgery with higher rate of complete resection is preferable to initial surgery. Radiotherapy maybe a feasible remedy to NSGCTs patients with residual tumors after chemotherapy and surgery. According to the results described above, our MDTs have reached a consensus that two to four courses of inducton chemotherapy (BEP) are used for the MMGCTs patients in the first-line treatment strategy. In the setting of disease measuring greater than 10 cm at diagnosis, adjuvant radiotherapy is recommended to patients who experience complete response. For patients with residual mass after neoadjuvant chemotherapy and delayed surgery, adjuvant radiotherapy consisting of boost to the residual mass is given. If the patients who experience incomplete response after chemotherapy are not performed surgical resection, definitive radiotherapy is administerd.

In summary, primary MMGCTs are rare and associated with adverse outcome compared with their gonadal counterparts. A multidisciplinary approach including chemotherapy, surgery, and radiotherapy is recommended to patients with MMGCTs. The role of radiation therapy in this setting merits further study.
